# Molecular dynamics and minigene assay of new splicing variant c.4298-20T>A of *COL4A5* gene that cause Alport syndrome

**DOI:** 10.3389/fgene.2023.1059322

**Published:** 2023-02-27

**Authors:** Lei Liang, Haotian Wu, Zeyu Cai, Jianrong Zhao

**Affiliations:** ^1^ Center for Prenatal Diagnosis and Medical Genetics, Affiliated Hospital of Inner Mongolia Medical University, Hohhot, China; ^2^ School of Public Health, Inner Mongolia Medical University, Hohhot, China; ^3^ Department of Nephrology, Affiliated Hospital of Inner Mongolia Medical University, Hohhot, China

**Keywords:** alport syndrome, COL4A5, splicing variant, minigene assay, molecular dynamics

## Abstract

**Introduction:** Alport syndrome (AS; OMIM#308940) is a progressive hereditary kidney disease characterized by hearing loss and ocular abnormalities. According to the mode of inheritance, AS has three subtypes: X-linked (XL; OMIM#301050), autosomal recessive (AR; OMIM#203780), and autosomal dominant (AD; OMIM#104200). XLAS is caused by a pathogenic variant in *COL4A5* (OMIM*303630) gene encoding type IV collagen (Col-IV) α5 chain, while ADAS and ARAS are consequences of a variant in *COL4A3* (OMIM*120070) and *COL4A4* (OMIM*120131) genes that encode Col-IV α3 and α4 chains, respectively. Usually, diagnosis of AS requires hereditary or pathological examinations. Splicing variants are hard to be determined as pathogenic or non-pathogenic based on the results of gene sequencing.

**Methods:** This study focused on a splicing variant in *COL4A5* gene, termed NM_000495.5: c.4298–20T>A, and to analyzed its authenticity and damaged α5 chain. In vitro minigene splicing assay was applied to investigate the effect of splicing variant, c.4298–20T>A, on *COL4A5* mRNA synthesis. Molecular dynamics method was used to predict the capability of the responsible α5(IV) to form a triple helix.

**Results:** The intron 46 of *COL4A5* mRNA retained 18 bp, resulting in insertion of six amino acids behind the amino acid at position 1,433 of α5(IV). The predicted protein effect of this variant: p. (Pro1432_Gly1433insAspTyrPheValGluIle). As a consequence, the stability of α5(IV) secondary structure was impaired, probably leading to the unusual configuration of α345(IV).

**Discussion:** Normally, splicing variant in *COL4A5* gene can lead to phenotypes of XLAS, and the effect is associated with the extent of splicing. The patient reported here carried a c.4298–20T>A splicing variant in COL4A5 gene, and AS was highly suspected based on the pathology results. However, the patient did not manifest any ocular or ear abnormalities. We therefore present the c.4298–20T>A splicing variant in *COL4A5* gene as likely-pathogenic splicing variant that leads to XLAS with mild phenotypes.

## 1 Introduction

Alport syndrome (AS) is a hereditary disorder accompanied by progressive kidney disease, sensorineural deafness, and ocular abnormalities (Nozu et al., 2019). *COL4A3*, *COL4A4*, and *COL4A5* encode type IV collagen (Col-IV) α3-α5 chains, respectively, while AS is caused by a defect in one of the three chains. According to the mode of inheritance, AS has three subtypes: X-linked (XLAS), autosomal recessive (ARAS), and autosomal dominant (ADAS). XLAS is a result of a variant in *COL4A5* gene encoding α5(IV). With advances in sequencing technology, more and more variants that may lead to aberrant splicing have been unraveled (Groopman et al., 2019; Yamamura et al., 2019). It is important to identify the pathogenicity of the variants, but in-vitro experimental data are required to confirm the underlying pathogenic principle (Nozu et al., 2014; Gao et al., 2020). Therefore, we believe that functional analysis, such as minigene splicing assay, can help further clarify the pathogenicity of the splicing variants of uncertain significance. The minigene splicing assay allows assessment for the effect of varying on splicing mechanism without the patient’s cellular sample. This study aimed at assessing the authenticity and potential effects of a newly identified splicing variant in *COL4A5* gene. We described the approaches capable of identifying the pathogenicity of splicing variants that may lead to AS, and we also introduced a new way to predict the effect of the variants in *COL4A5* gene on α345(IV) trimerization using molecular dynamics.

## 2 Materials and methods

### 2.1 Ethical compliance and patient information

A 15-year-old male patient from the affiliated Hospital of Inner Mongolia Medical University, from the Chinese mainland, Hohhot, Inner Mongolia Autonomous region, Han nationality, denied consanguineous marriage. He had no family history of renal disease. The patient’s parents and five-year-old brother have normal phenotypes. This study was approved by the Ethics Committee of the affiliated Hospital of Inner Mongolia Medical University and the patients’ written informed consent was obtained. The patient was admitted to the hospital for half a month because of urinary protein. The abdomen is soft without tenderness, the liver and spleen are not reached, there is no percussion pain in both kidney areas, and there is no edema in both lower limbs. Auxiliary examination: urinary protein 3+, 24-h urinary protein,3.53g/24 h, blood uric acid 491umol/l. The patient showed no hearing loss with an auditory test or ocular abnormalities on examination by an ophthalmologist. Color Doppler ultrasound of urinary system: increased light spots in bilateral renal cortex. The renal biopsy, mainly the renal cortex, was routinely stained with HE, PAS, PASM, and Masson. A striped gray-yellow tissue was fixed with neutral glutaraldehyde and was taken out of a box (0.5 cm) for electron microscope examination. His renal EM findings showed irregular thinning and thickening, as well as a diffuse basket-weave pattern that suggested highly suspected presence of AS.

### 2.2 Whole exome sequencing and sanger sequencing

Peripheral blood of broadband was collected by EDTA anticoagulant tube. The genomic DNA extraction was undertaken with the Blood Genomic DNA Extraction Kit (TIANGEN, China). Library preparation and exome capture were performed with Hieff NGS®OnePot DNA Library Prep Kit for Illumina® (YEASEN, China) and xGen Exome Research Panel v1.0 (Integrated DNA Technologies, Inc., United States). Captured libraries were sequenced on HiSeq2500 (Illumina, California, CA, United States).

The quality of the raw data was checked by FASTQC. The clean reads were mapped to the reference genome (GRCh37/hg19) with BWA software. After removing duplicates and base quality recalibration, SNP and Indel variants were called using the GATK pipeline. Detected variants were annotated using ANNOVAR and filtered with minor allele frequencies (MAFs) of <0.5% for dbSNP, 1000G, ExAC, gnomAD databases. Additionally, Copy number variation analysis was performed of probe coverage regions using xhmm and clamms tools.

The interpretation rules of sequence variation data refer to the American College of Medical Genetics and Genomics (ACMG) genetic variation classification standards and guidelines, ClinGen sequence variation interpretation (SVI) and the guidelines for specific genes and diseases.

In order to verify the variant, Peripheral blood DNA from the proband was subjected to Sanger sequencing. The PCR conditions were as follows: a pre-denaturation step (95°C for 5 min), followed by 35 cycles of denaturation (95°C for 30 s), annealing (60°C for 30 s), elongation (72°C for 30 s), with a final extension (72°C for 5 min). The amplicon was sequenced on the ABI 3730xl Genetic Analyzer (Applied Biosystems, Inc.). Sequences were compared using CodonCode Aligner to reference sequences.

### 2.3 Construction of in vitro expression vector

The wild-type and mutant type plasmids were constructed based on the mutation: *COL4A5*: c.4298–20T>A. The cloning vector is pMini-CopGFP, and the clone site is BamHI/XhoI. The normal genomic DNA and the genomic DNA carries *COL4A5*: c.4298–20T>A mutation were amplified by seamless primers. The wild-type and mutant target gene fragments were obtained and inserted into the cloning vector by double enzyme digestion and recombination reaction. The recombinant product was transformed into competent cells and cultured, and the clones were selected for PCR amplification. Sanger sequencing was carried out to determine whether the target fragment of the *COL4A5* gene was correctly inserted into the vector. The correct recombinant wild-type minigene plasmid and mutant minigene plasmid were selected.

### 2.4 RT-PCR, PCR and sequencing

The plasmid was extracted and transfected into 293T cells after culturing the bacterial solution of 5–15 ml. RNA reverse transcription cDNA was extracted from the transfected cells. Primers were designed for RT-PCR amplification and gel electrophoresis.

### 2.5 The evolutionary conservation analysis of amino acid residues and the structure prediction of mutant proteins

The protein sequences of different species were downloaded from NCBI. Jalview software was used for multiple sequence alignment and conservation analysis. The I-TASSER server was used to simulate the influence of the variable region. The three-dimensional structure of the protein was visualized by PyMol software.

### 2.6 3-D structure analysis of α345(IV)

The 3-D structure of the α345(IV) wild-type trimmer was generated *via* homology modeling through MODELLER software. PDB IDs 3HQV, 5NB0, 5NB1, and 5NAZ were chosen as templates for the triple helical region and the NC1 domains of α3(IV)–α5(IV), respectively. The 3-D structure of c.4298–20T>A mutant α5(IV) and its mutant trimmer were prepared from the amino acid sequences of wild-type α5(IV). The resulting structures were structurally optimized by GROMACS version 2020.4 (http://manual.gromacs.org). The AMBER99SB force field was used for all simulations.

### 2.7 Molecular dynamics

GROMACS software package was used to simulate the protein molecular dynamics. The protein uses the AMBER14sb force field. The protein was loaded into the GROMACS module, and hydrogen atoms and NaCl ions were added. Select TIP3P dominant water model and set periodic boundary conditions. The workflow of molecular dynamics simulation includes four steps: energy minimization, NVT equilibrium, NPT equilibrium and production dynamics simulation. Firstly, the protein heavy atoms were constrained to minimize the energy of water molecules by 10,000 steps (including 5,000 steps steepest descent method and 5,000 steps conjugate gradient method); Then, maintaining the constraints, 50,000 step NVT ensemble simulation was carried out for the whole system. The temperature was 298K, and the time step was 2fs; Then 50,000 step NPT ensemble simulation was carried out for the whole system, the temperature was 298k, and the time step was 2fs; Finally, the molecular dynamics simulation of the system was carried out in the NPT ensemble for 100ns with a time step of 2fs. The relevant parameters were analyzed by the module of the GROMACS software package.

## 3 Results

### 3.1 Pathologic diagnosis

**FIGURE 1:**
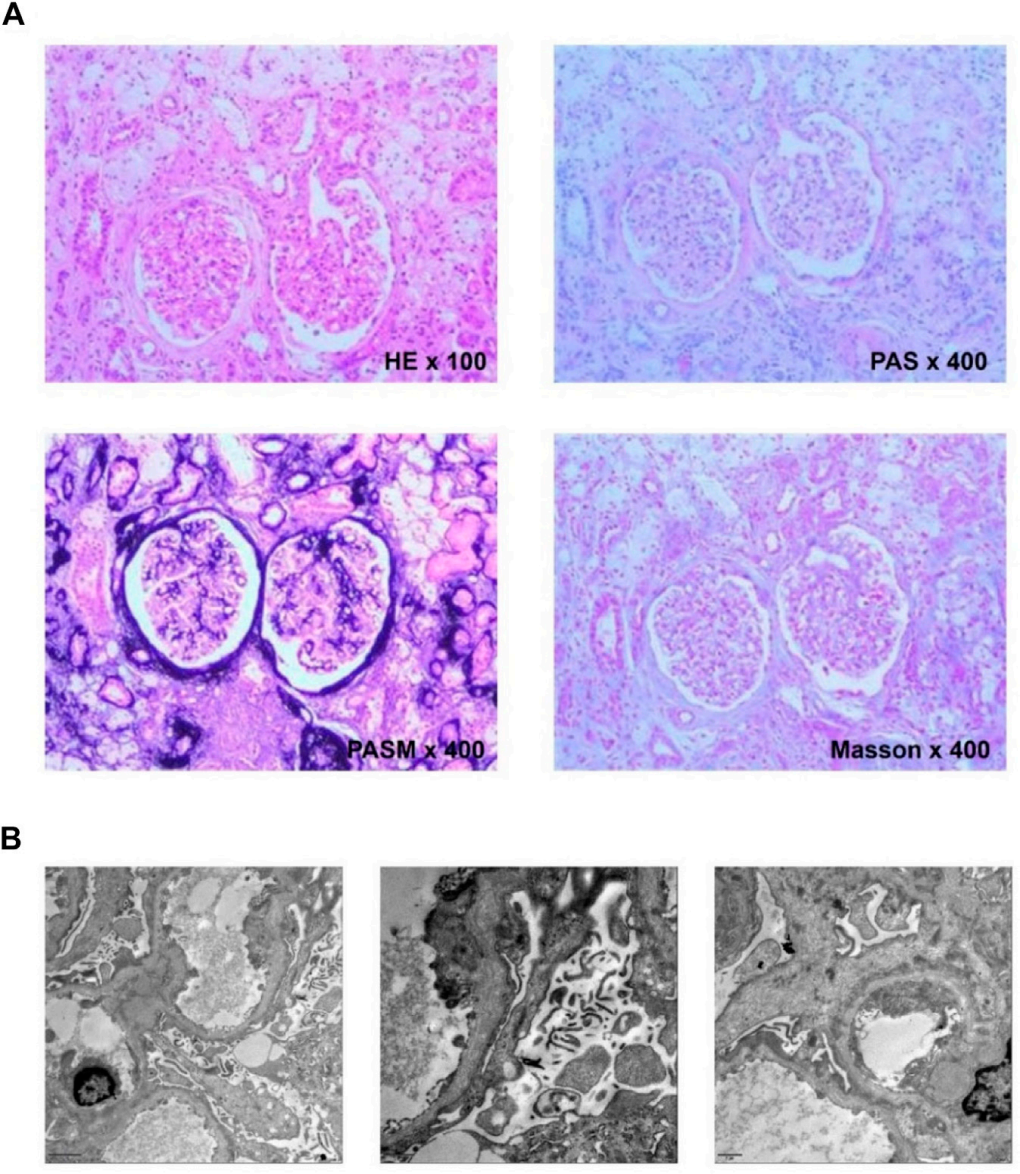
**(A)** Histological examination. HE showed granular degeneration of renal tubular epithelial cells and focal foam-like cell infiltration; PAS showed sclerotic glomeruli and foam-like cell infiltration next to scleroses glomeruli; PASM showed heterogeneous staining of the capillary loop basement membrane; Masson showed no obvious deposition of the furophilic protein in the glomeruli. **(B)** Ultrastructural. The basement membrane is torn and showed cobweb-like structures in all three pictures of the electron microscope.

The tissue was obtained by a renal puncture, and a routine renal pathological examination was performed. HE showed 10 glomeruli, two of which had glomerular sclerosis. Six glomeruli were seen by PAS, PASM and Masson staining, including 1 with glomerular sclerosis. No obvious endothelial cell proliferation and deposition of furoglobin in the mesangial region were seen. The capillary loop was open, and the basement membrane was slightly thickened. There were no nail process-like structures, no mesangial insertion, double-track formation, no obvious deposition of hemophiliac protein under the epithelium and endothelium, no obvious proliferation of parietal epithelial cells, and no crescent formation. The capillary loop basement membrane showed uneven staining (Figure 1A). The thickness of the basement membrane was about 200–600 nm. The dense layer of the segmental basement membrane was thickened, and some of them were torn and showed cobweb-like structures (Figure 1B).

### 3.2 Identification of COL4A5 mutation

**FIGURE 2:**
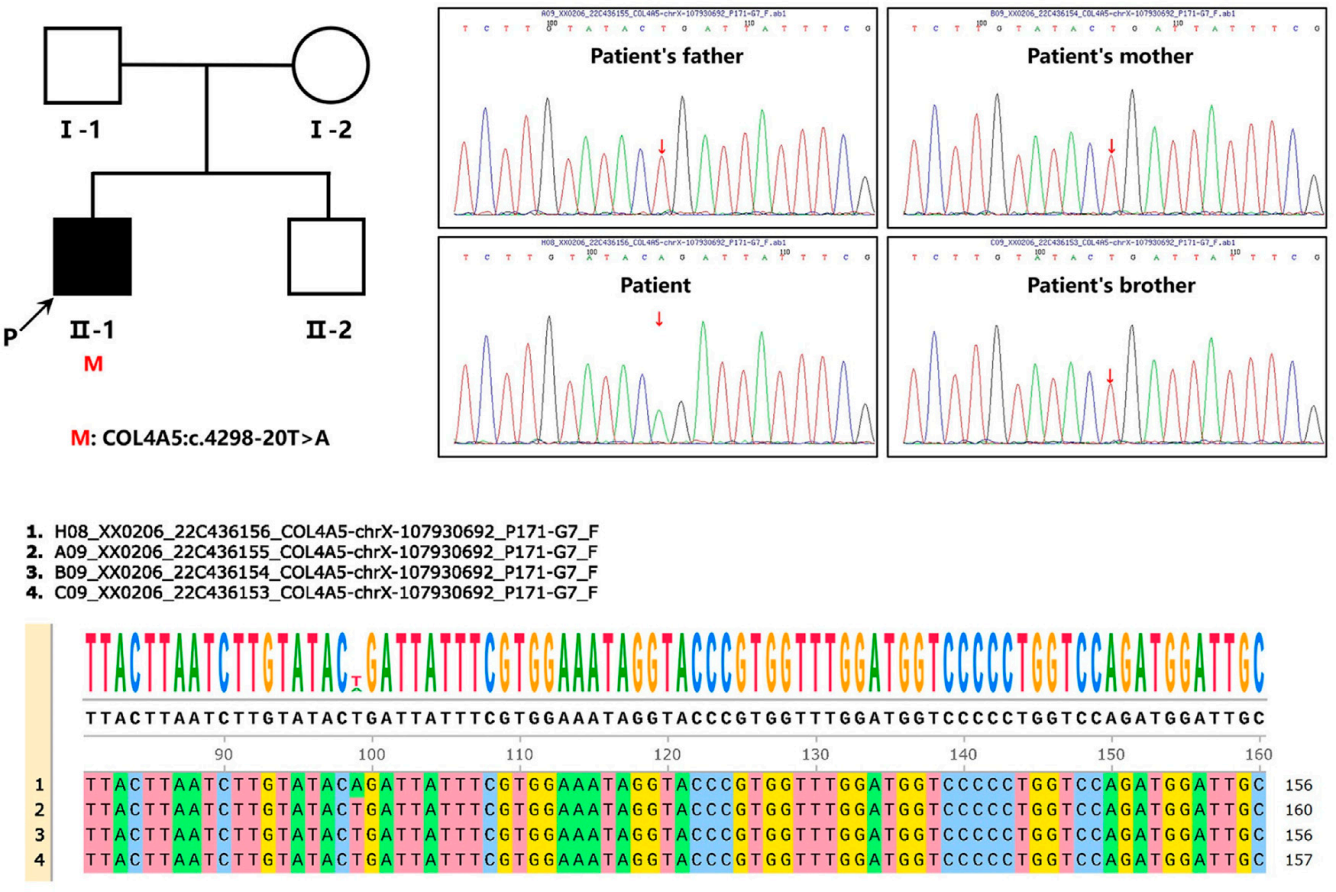
Pedigree and sequencing of patient from one unrelated Chinese family. An arrow points to the patient involved in this study. Analysis of the genomic DNA of the designated patient, his parents, and his five-year-old brother by Sanger sequencing. The gene variation is shown by a red arrow.

We found c.4298–20T>A mutation in the patient’s *COL4A5* gene. The mutation is not included in the gnomAD East Asian population database and has not been reported in the literature. Neither the patient’s parents nor his five-year-old brother exhibit any clinical signs of kidney disease. Each of them underwent Sanger sequencing to determine whether they carried the same mutation as the patient’s *COL4A5* gene (Figure 2). In light of the verification results of Sanger sequencing in the patient family, it can be concluded that the variant carried by the patient is a *de novo* mutation (Figure 2).

### 3.3 Corroboration of COL4A5 mutations as the determinant of alternative splicing in splicing assay

**FIGURE 3:**
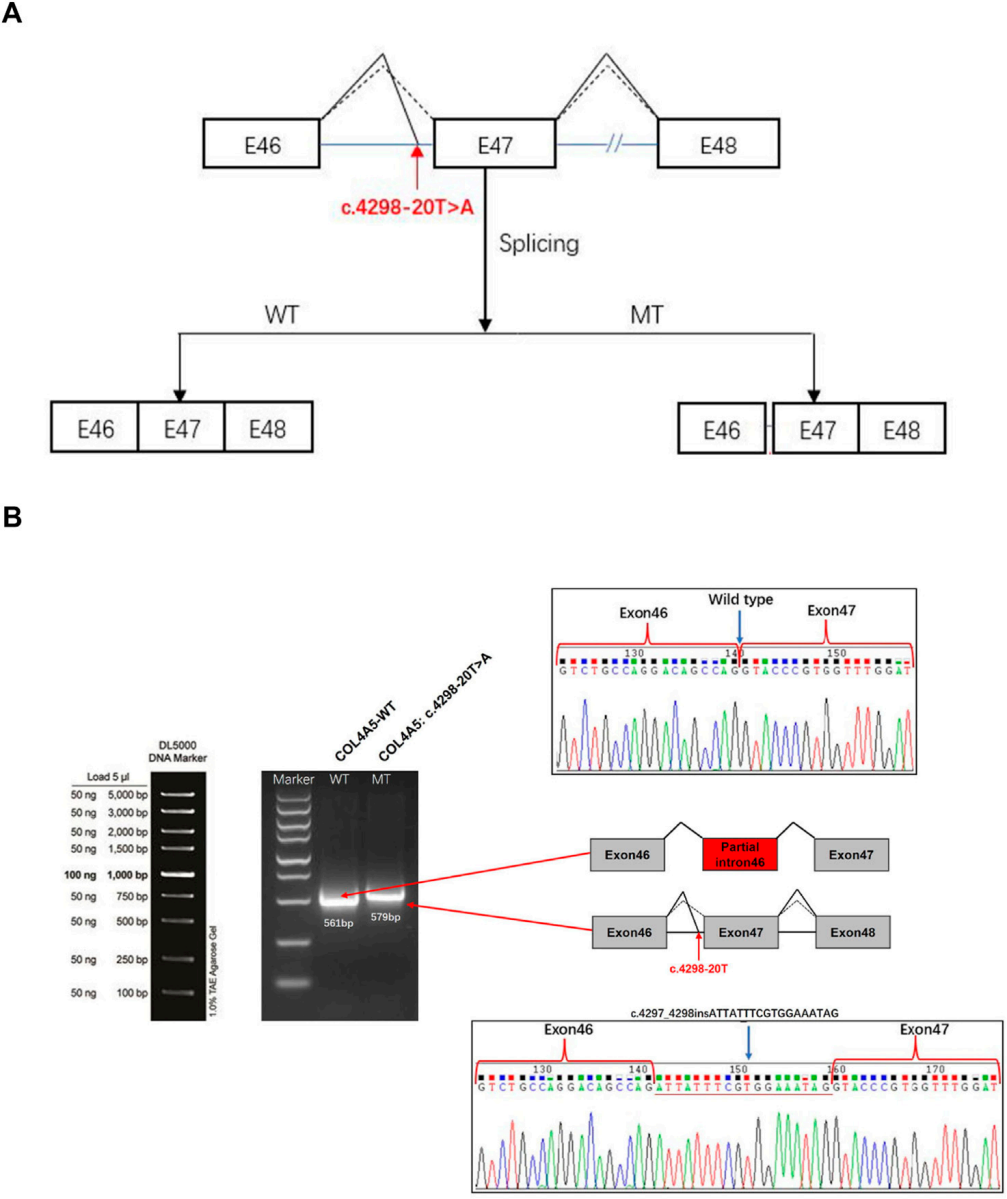
*COL4A5* gene constructs for splicing pattern investing of the patient. **(A)** Splicing schematic diagram. The Bioinformatics software dbscSNV and SpliceAI predicted that the mutation might lead to a splicing variation of exon 46 of the *COL4A5* gene. **(B)** *COL4A5* gene c. 4298–20T>A splicing pattern Minigene verification experiment. *COL4A5*-WT and *COL4A5*-MT were transiently introduced into 293T cells. After the RNA was extracted, the splicing products were analyzed by RT-PCR. Lower bands represent correct splicing, while higher bands represent the *COL4A5* inserted 18 nucleotides in intron 46.

We used bioinformatics software dbscSNV and SpliceAI to analyze c.4298–20T>A. Our results suggested that the mutation may lead to the intron 46 of *COL4A5* mRNA retained (Figure 3A). We constructed the wild-type (WT) and mutant (MT) plasmids targeting *COL4A5*: c.4298–20T>A mutation and then transfected them into 293T cells. Next, RNA was extracted for reverse transcription, and cDNA was amplified by PCR. The abnormal splicing of the mutant mRNA was verified by analyzing the band size of PCR amplification products and Sanger sequencing results. The results showed that the transcribed mRNA sequence of the WT plasmid was as expected, and it contained a complete mRNA product transcribed by exon46 and exon47; the sequence of the mutant plasmid showed that the 18bp of intron46 was retained and the alteration of c.4298–20T>A (c.4297_4298insATTATTTCGTGGAAATAG) affect mRNA splicing (Figure 3B).

### 3.4 Model analysis of abnormal structure of COL4A5 caused by mutation

**FIGURE 4:**
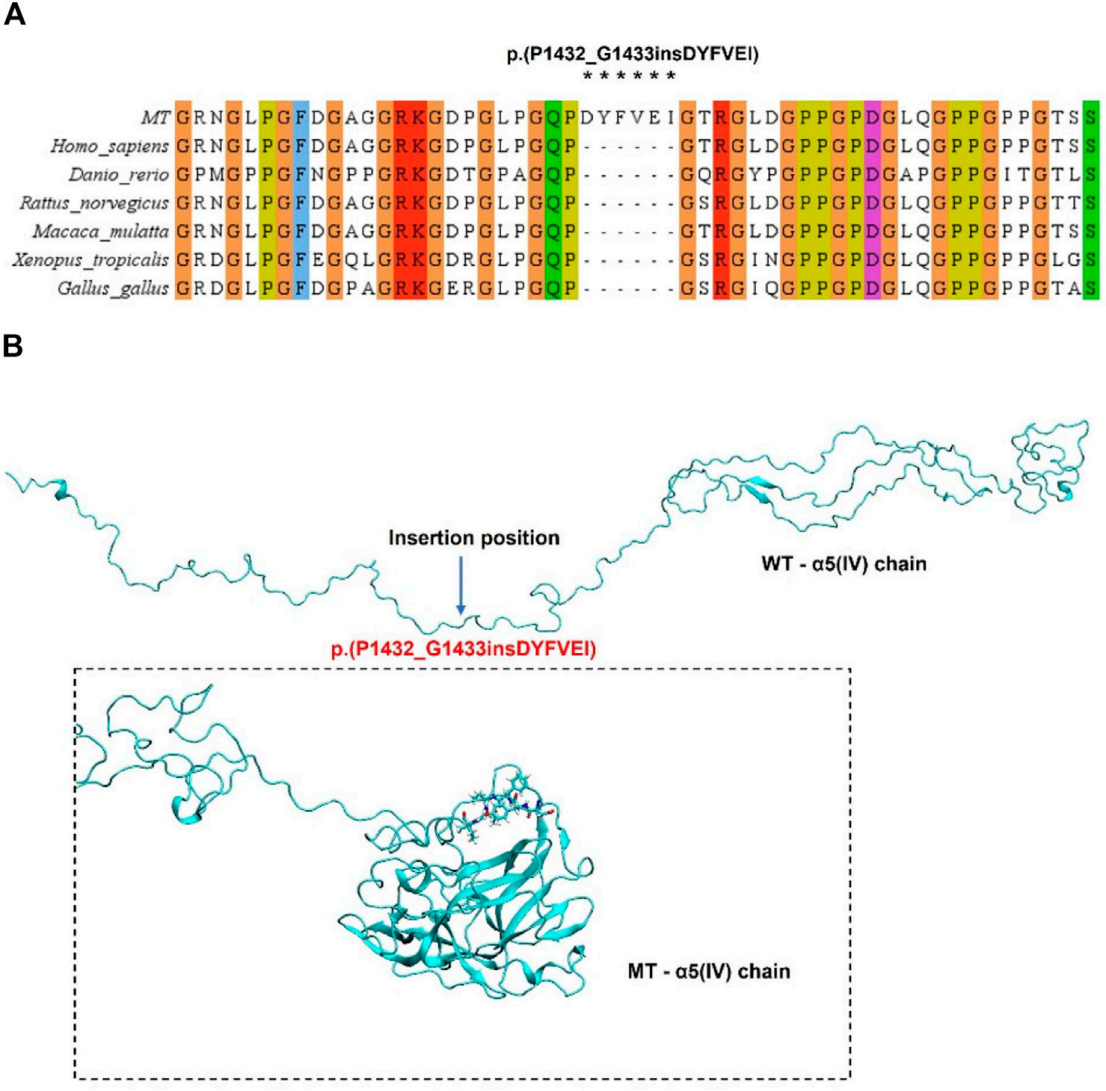
Analysis of *COL4A5* mutations. **(A)** Evolutionary conservation of amino acid residues altered by p.(P1432_G1433insDYFVEI) across different species. NCBI accession numbers are *Homo sapiens*: NP_000486.1; *Danio rerio*: NP_001116702.1; *Macaca mulatta*: XP_014983488.2; *Xenopus tropicalis*: XP_004916922.2; *Gallus gallus*: XP_015134092.2. Asterisk (*) means Inserted amino acids. **(B)** Through homology modeling analysis, the blue label is the magnification to confirm the Inserted amino acids at positions 1,432–1,433.

The evolutionary conservation analysis of the damaged domain by the in-frame insertion revealed a highly evolutionarily conserved among different species (Figure 4A). We used i-tasser and PyMOL software to predict the structure of WT and mutant *COL4A5* protein and build models. The complete *COL4A5* WT amino acid sequence (*COL4A5*: NM_000495.5) was used to construct the model, and the mutation site was c.4298–20T>A, which led to the change of mutant amino acid sequence to p.(P1432_G1433insDYFVEI), that is, six additional amino acids were inserted between P1432 and G1433. Compared with *COL4A5* wild type, the overall spatial structure of *COL4A5* mutant changed significantly. After the insertion of DYFVEI part into the WT chain, the number of the β-sheet and α-helix in the MT chain increased. Specifically, the number of sheet structure has increased from 10 to 100 and α-helix increased from 42 to 90. The whole MT chain starts to collapse (Figure 4B).

### 3.5 3-D structure analysis of the α5(IV) and α345(IV) trimer

**FIGURE 5:**
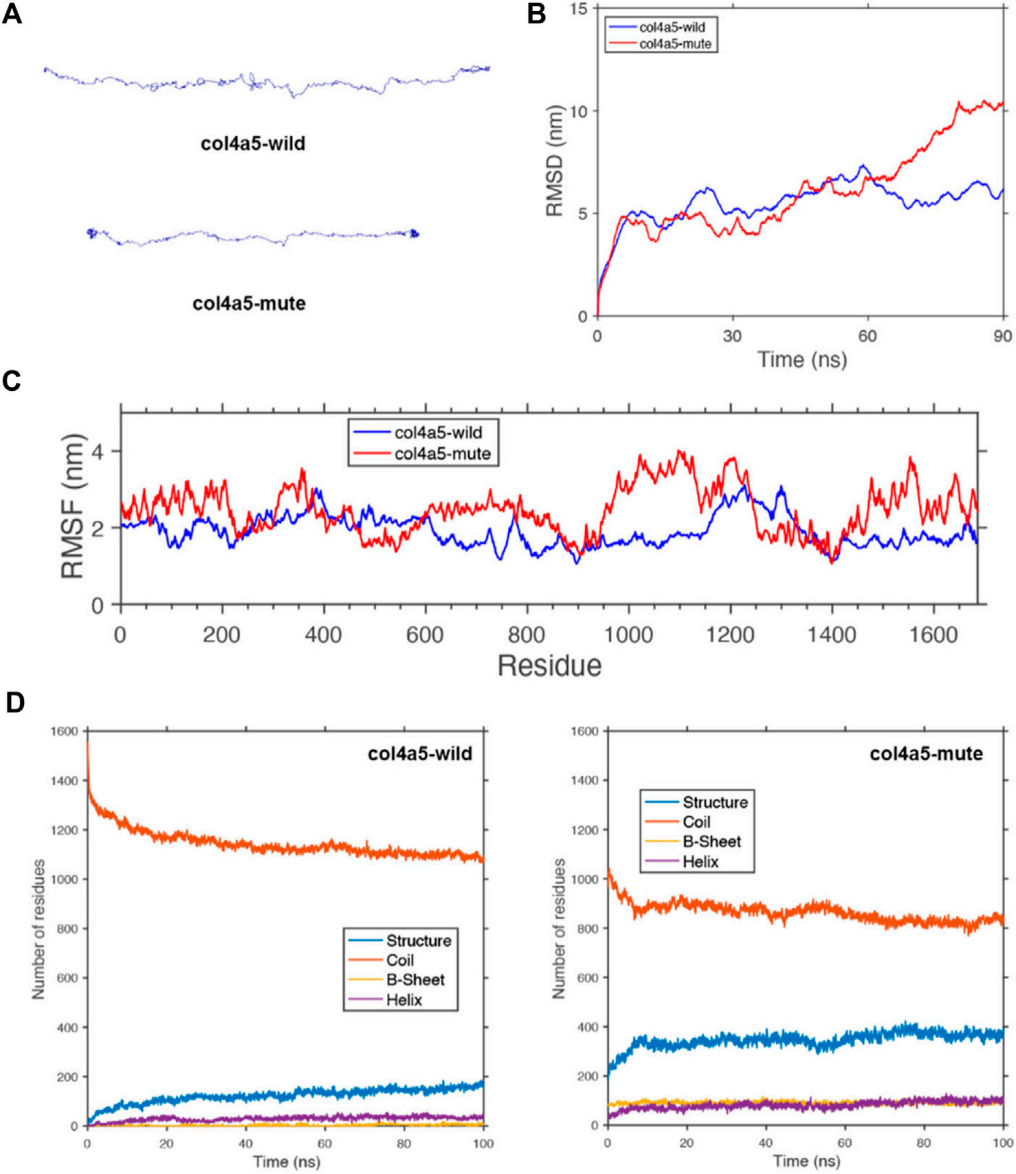
Protein molecular dynamics simulation of the α5(IV). **(A)** The conformation of the *COL4A5*-wild and -mute chains through molecular dynamics simulations at 90 ns. **(B)** RMSD of *COL4A5*-wild and -mute chains during 90 ns. **(C)** RMSF of each amino acid of the *COL4A5*-wild and -mute chains from 80 ns to 90 ns. **(D)** The numbers of the secondary structures of the *COL4A5*-wild and -mute chains during 100 ns.

**FIGURE 6:**
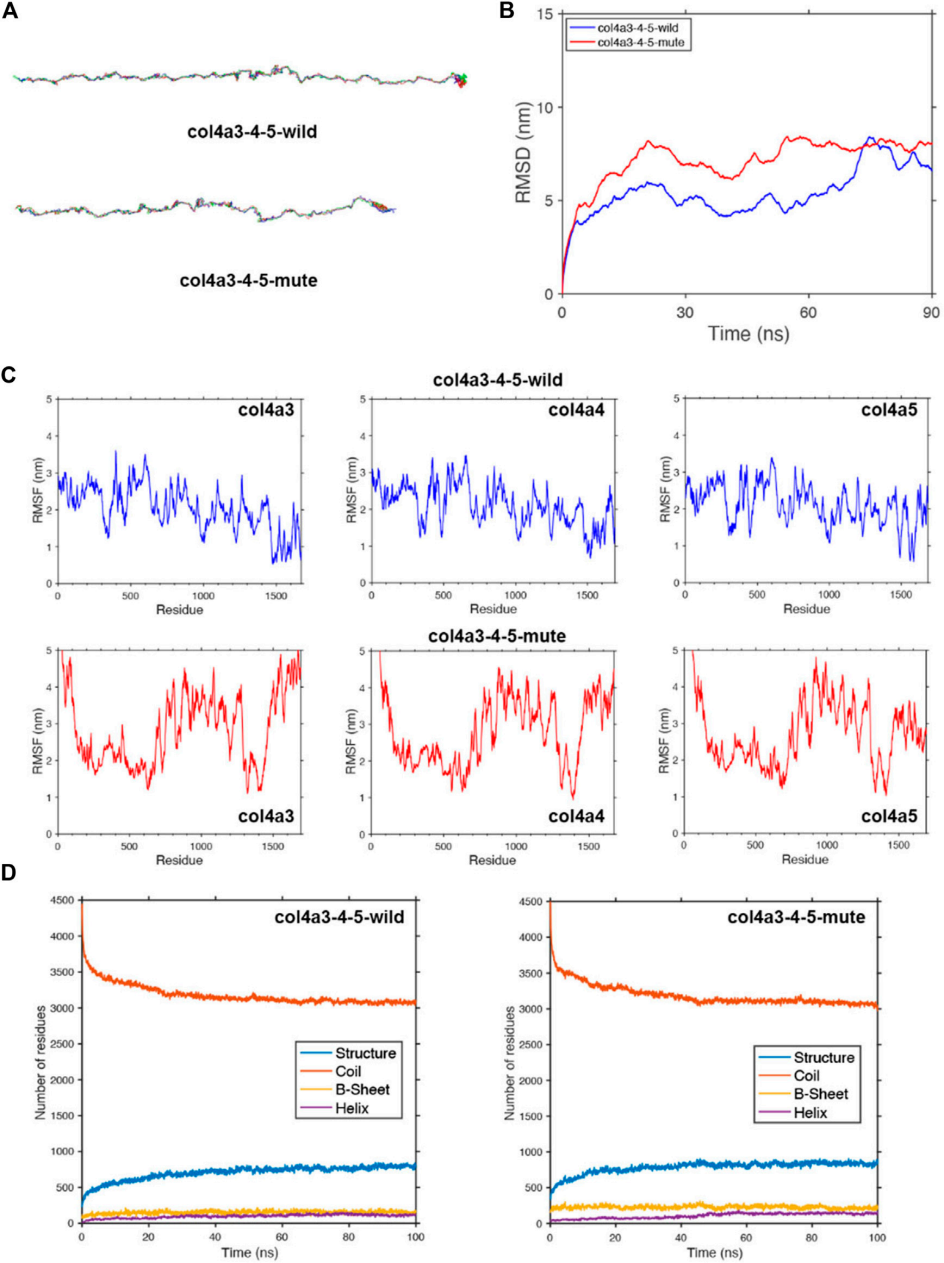
Protein molecular dynamics simulation of the α345(IV) trimer. **(A)** The conformation of the α345(IV)-wild and -mute triple chains through molecular dynamics simulations at 90 ns. **(B)** MSD of α345(IV)-wild and -mute chains during 90 ns. **(C)** RMSF of each amino acid of the α345(IV)-wild and -mute chains from 80 ns to 90 ns. **(D)** The numbers of the secondary structures of the α345(IV)-wild and -mute chains during 100 ns.

We use homology modeling to generate the three-dimensional structure of the α5(IV) and α345(IV) trimer. The 3-D structure of the α5(IV) wild- and mute-type chains were presented as an amino acid type. The α5(IV) constructed by p.(P1432_G1433insDYFVEI) was found to aggregate, leading to a lower total potential energy (Figure 5A). The 3-D structure of the α345(IV) wild- and mute-type trimers were presented as ribbon type. Formation of α345 (IV) trimer following p.(P1432_G1433insDYFVEI), with the structure not completely collapsed (Figure 6A).

### 3.6 Protein molecular dynamics simulation of the α5(IV) and α345(IV) trimer

To study the geometric structure deviation of the wild-type (WT) and mute-type (MT) *COL4A5* chains, we used in full atom molecular dynamics simulation to conduct a 100 ns kinetic study. RMSD results show that the WT chain achieved the equilibrium structure more quickly (about 30 ns), while the MT chain needed a longer time (about 80 ns) to reach the equilibrium structure (Figure 5B). This is due to the change of the amino acids of the MT chain, which can be further seen from the following RMSF characterization. The heights of the RMSF values, describing the fluctuation of the corresponding amino acid residue, show that the fluctuation of the 1,430–1,600 residues in the MT chain significantly increased due to the insertion of DYFVEI part as well as the fluctuation of the 900–1,150 residues in the MT chain (Figure 5C). Comparing the changes of different secondary structure numbers in the MT and WT chains, the number of the β-sheet and α-helix in the MT chain increased. Specifically, the number of sheet structure has increased from 10 to 100 and α-helix increased from 42 to 90 (Figure 5D).

To study the geometric structure deviation of the wild-type (WT) and mute-type (MT) COLA345 triple chains, we used in full atom molecular dynamics simulation to conduct a 100 ns kinetic study. RMSD results show that the WT chain achieved the equilibrium structure more quickly (about 30 ns), while the MT chain needed a longer time (about 50 ns) to reach the equilibrium structure (Figure 6B). This is due to the change of the amino acids of the MT chain, which can be further seen from the following RMSF characterization. The heights of the RMSF values, describing the fluctuation of the corresponding amino acid residue, show that the fluctuation of the head and end parts as well as the 700–1,400 residues in the MT chain significantly increased due to the insertion of DYFVEI part (Figure 6C). Comparing the changes of different secondary structure numbers in the MT and WT chains, the number of the β-sheet and α-helix in the MT chain increased. Specifically, the number of sheet structure has increased from 160 to 230 and α-helix increased from 100 to 120 (Figure 6D).

## 4 Discussion

It is well established that genotype-phenotype shows a strong correlation in XLAS (Jais et al., 2000; Gross et al., 2002; Bekheirnia et al., 2010). Jais *et al* reported that large deletions and non-sense mutations confer a 90% probability of end-stage renal disease (ESRD) by the age of 30 years old compared with a 70% risk with splice site mutations and a 50% risk with missense mutations (Jais et al., 2000). Gross *et al* grouped men with XLAS into three groups as follows (Gross et al., 2002). 1) Large rearrangements, frame shift, non-sense, and splice donor site mutations had a mean ESRD age of 19.8 ± 5.7 years. 2) Non-glycine or 3’ glycine missense mutations, in-frame deletions/insertions, and splice acceptor site mutations had a mean ESRD age of 25.7 ± 7.2 years, and 3) 5’ glycine substitutions had an even later onset of ESRD at a mean of 30.1 ± 7.2 years (Gross et al., 2002). Bekheirnia *et al* reported the average onset of ESRD as 37 years old for those with missense mutations, 28 years old for those with splice site mutations, and 25 years old for those with truncating mutations (Bekheirnia et al., 2010). Although these reports very clearly show genotype-phenotype correlations, all studies have grouped splice site mutations together without considering their diverse consequences for collagen transcripts. Kandai Nozu *et al* reported that 29% of men with XLAS showed significantly milder phenotypes, including milder proteinuria, later onset of ESRD, and less occurrence of hearing loss (Hashimura et al., 2014). All of them had non-truncating mutations. From these results, it was suggested that in-frame mutations could show a milder phenotype, even if derived from a splice site mutation (Hashimura et al., 2014). In this study, our patient had persistent haematuria, mild proteinuria, no sensorineural deafness and no ocular abnormalities, thus making for a relatively mild phenotype of male XLAS. However, the EM findings of the glomerular basement membrane (GBM) showed the typical abnormalities for AS such as irregular thinning and thickening and a diffuse basket-weave pattern, leading to a diagnosis of AS. Then we found an exon 47 c.4298–20T>A variant in *COL4A5* gene in a patient. This variant is not included in the gnomAD database and has not been verified *in vitro* yet. Neither the patient’s parents nor his five-year-old brother exhibit any clinical signs of kidney disease. The results of their Sanger sequencing confirmed their *COL4A5* gene was wild-type and did not have the same mutation as the patient. We visited several bioinformatics platforms, including SpliceAI, dbscSNV_ADA, dbscSNV_RF, and varSEAK, to analyze the effect of the variant on the primary splicing site. Consistently, unusual splicing in the *COL4A5* gene was suggested upon the occurrence of the variant. However, it is challenging to accurately identify the abnormal splicing sites and transcripts with a bioinformatics platform at all times. Therefore, for the variants that may affect splicing site and are detected by next-generation sequencing (NGS), it is significant to validate their authenticity (Malone et al., 2017; Horinouchi et al., 2019). With transcript analysis, we can determine splicing site variant as either truncating or non-truncating variant, which will help with future analysis of genotype-phenotype correlations.

We applied an *in vitro* minigene splicing assay to detect the aberrant splicing caused by a variant in the *COL4A5* gene. This method can easily detect the aberrant exonic or intronic splicing caused by a single-base substitutions (Fu et al., 2016; Horinouchi et al., 2020). Here, we adopted this method in a XLAS case with a single-base substitutions caused by the c.4298–20T>A variant in the *COL4A5* gene. Transcript analysis was not available in this case due to the low peripheral expression of *COL4A5* gene. Thus, we established a vector to carry the promoter, exon 46, and exon 47 of *COL4A5* gene. This vector was introduced to the prepared cells, and the transcripts produced were processed for reverse transcription–polymerase chain reaction (RT-PCR). The result demonstrated that the c.4298–20T>A variant preserved 18 bp from the intron 46 of *COL4A5* transcripts, resulting in insertion of six amino acids behind the amino acid at position 1,432 in α5(IV). Collectively, the non-coding sequences in the eukaryotic genome are composed of the non-coding regions and introns. The introns are removed during mRNA processing. Thus, no non-coding sequence (introns) is present in mature mRNA. In addition, the introns are non-sense for the structure of translation products and are free from the pressure of natural selection. Therefore, they are more prone to develop variants than exons. In the original and updated ACMG/AMP guidelines, no non-coding sequence variant (other than the typical splicing site variants) or splicing defect with deletion of one or more exon is documented, since the pathogenicity of no non-coding sequence variant is difficult to identify without experimental data. In the present study, the 18bp non-coding sequences of the intron46, which should be removed during normal transcription, were retained after a c.4298–20T>A variant in *COL4A5* gene and then converted to coding sequences (exons). Therefore, we reassessed the pathogenicity of the c.4298–20T>A variant according to the ACMG guidelines. PVS1_Moderate: this splicing region variant results in a new splicing acceptor in intron 46, leading to a non-frame shift insertion of 18 bp (6 aa) at the beginning of exon 47; PP4: the clinical phenotypes were highly consistent with the single-gene hereditary disease caused by *COL4A5* gene abnormalities; PP3: Bioinformatics software predicted the potential effect of the variant on gene splicing; PS3_Moderate: functional analysis demonstrated that the variant affected gene splicing, resulting in intron retention (18 bp); PM2_Supporting: the variant is rare and is not included in the gnomAD database. Combining the results, the base insertion caused by the c.4298–20T>A was assessed as likely-pathogenic.

α3(IV), α4(IV), and α5(IV) form a triple helix that combines tightly with other triple helices to form the GBM. If one of the three α chains becomes defective from a pathogenic variant of the encoding gene, the normally highly ordered GBM gradually breaks down, including the splitting of the lamina densa in GBM, which is referred to as the basket weave change. These changes accelerate the glomerular sclerotic changes and lead to kidney dysfunction. XLAS is caused by α5(IV) chains following a pathogenic variant in the protein-coding gene. Upon a mutation in the *COL4A5* gene, there are two following cases: 1) complete loss or shortening of the α5-chain protein product; 2) a full-length protein product with amino acid substitution or insertion. The former case is easy to understand, as incomplete protein may not function normally thereby resulting in diseases. Additionally, it is generally believed that amino acid substitutions/insertions can lead to local kinks or abnormal folding without a triple helix structure, while the abnormally folded collagen molecules have increased sensitivity to protease, making them prone to degradation (Kamura et al., 2020). Therefore, the effect of *COL4A5* gene mutations on the folding of its triple helix structure is critical to the severity of clinical phenotypes. Usually, a strong genotype-phenotype relationship is presented in male XLAS cases (Jais et al., 2000; Bekheirnia et al., 2010; Kamiyoshi et al., 2016). In terms of truncation variations (e.g., non-sense variation, and deletion/insertion), male XLAS cases present with complete negative expression of α5(IV), while female XLAS cases show chimeric α5(IV) expression (Jais et al., 2003; Said et al., 2017; Yamamura et al., 2017). For some non-truncation pathogenic variants (e.g., missense variation), α345(IV) trimer with a structure (not exactly matched with the normal structure) can be formed, and the α5(IV)-positive patients present with milder phenotypes than α5(IV)-negative patients (Hashimura et al., 2014; Imafuku et al., 2018). Splicing variants are more complex, commonly including deletion of exon (in whole or in part), retention of intron in whole (conversion from intron to exon), retention of intron in part (splicing happens in the sites, other than the splicing sites, with splicing site features in intron), depletion of multiple exons. These aberrant splicing variants can lead to consequences, such as deletion of amino acids, premature translation termination, and insertion of multiple amino acid sequences like the case reported in this study. To clarify the genotype-phenotype relationship in this patient, we applied to molecular dynamics simulation to analyze the effect of the *COL4A5* gene variation on the capability of protein to form a triple helix (Salomon-Ferrer et al., 2013). Homology modeling was employed to construct a three-dimensional structure for the α345 (IV) trimer.

The *COLA45* gene contains 51 exons. The sizes of exons of *COL4A5* (5'- and 3’- untranslated sequences not included) vary between 27 and 213 bp. Exon one contains 283 bp with 202 bp of a 5'-untranslated sequence and 81 bp of a translated sequence. The translated sequence of exon one encodes solely the tentative 26-residue long signal peptide. Exon two encodes the 14 non-collagenous amino-terminal end and two Gly-X-Y triplets. Thus, exons 2–47 encode the collagenous domain, exon 47 being a junction exon encoding the carboxyl-terminal end of the collagenous domain and a part of the non-collagenous domain. In our study, c.4298–20T>A variant preserved 18 bp from the intron 46 of *COL4A5* transcripts, resulting in insertion of six amino acids behind the amino acid at position 1,432 in wild α5(IV), that is, the additional DYFVEI added between exon 46 and exon 47 is located at the tail of the α5 chain. We carried out full atom molecular dynamics simulations of the mutant α5 chain *via* a 100 ns kinetic simulation process. RMSD and RMSF results show that the fluctuation of the 1,430–1,600 residues in the mutant α5 chain significantly increased due to the insertion of DYFVEI part as well as the fluctuation of the 900–1,150 residues in the mutant α5 chain. The molecular dynamics results show that the c.4298–20T>A variant affects not only the tail stability of the α5 chain, but also the stability of the middle part of the α5 chain. Then we carried out full atom molecular dynamics simulations of the mutant α345(IV) trimer *via* a 100 ns kinetic simulation process. RMSD and RMSF results show that the fluctuation of the head and end parts as well as the 700–1,400 residues in the mutant α345(IV) trimer significantly increased due to the insertion of DYFVEI part. Molecular dynamics results showed that the c.4298–20T>A variant affected not only the tail stability of α345(IV) trimer, but also the stability of the head and middle of α345(IV) trimer. With this method, the triple helix structure of mutant α345(IV) was well simulated, and the changes in the trimer were monitored with the variant information. Taken together, these simulations suggest that the mutant α345(IV) trimer following an intron 46 splicing variant in *COL4A5* gene, with the structure not completely collapsed, but the structure of mutant α345(IV) trimer changes greatly, and mutation has a great impact on their configuration. Whether molecular dynamics simulation can help reflect the clinical severity of pathogenic variants is now in progress in our laboratory.

## 5 Conclusion

To conclude, we identified a splicing variant c.4298–20T>A in *COL4A5* gene in a XLAS patient. Through histopathological examination of the kidneys, we provided *in vivo* evidence for the pathogenicity of the variant and expanded the variant spectrum of *COL4A5* gene under an X-linked dominant mode of inheritance. The findings of the study help increase our understanding of the molecular pathogenesis of AS. Meanwhile, as NM_000495.5: c.4298–20T>A variant is currently cited as “likely pathogenic” change in ClinVar (https://www.ncbi.nlm.nih.gov/clinvar/RCV001281232/) and dbSNP (rs2068567564) databases, our study strongly supports experimentally a pathogenic effect.

## Data Availability

The data presented in the study are deposited in the Genome Sequence Archive in National Genomics Data Center, accession number HRA003934.
